# Using Sparse Patch Annotation for Tumor Segmentation in Histopathological Images

**DOI:** 10.3390/s22166053

**Published:** 2022-08-13

**Authors:** Yiqing Liu, Qiming He, Hufei Duan, Huijuan Shi, Anjia Han, Yonghong He

**Affiliations:** 1 Institute of Biopharmaceutical and Health Engineering, Tsinghua Shenzhen International Graduate School, Shenzhen 518055, China; 2 Department of Pathology, The First Affiliated Hospital, Sun Yat-sen University, Guangzhou 510080, China

**Keywords:** histology images, tumor segmentation, sparse annotation, weakly-supervised learning, semi-supervised learning

## Abstract

Tumor segmentation is a fundamental task in histopathological image analysis. Creating accurate pixel-wise annotations for such segmentation tasks in a fully-supervised training framework requires significant effort. To reduce the burden of manual annotation, we propose a novel weakly supervised segmentation framework based on sparse patch annotation, i.e., only small portions of patches in an image are labeled as ‘tumor’ or ‘normal’. The framework consists of a patch-wise segmentation model called PSeger, and an innovative semi-supervised algorithm. PSeger has two branches for patch classification and image classification, respectively. This two-branch structure enables the model to learn more general features and thus reduce the risk of overfitting when learning sparsely annotated data. We incorporate the idea of consistency learning and self-training into the semi-supervised training strategy to take advantage of the unlabeled images. Trained on the BCSS dataset with only 25% of the images labeled (five patches for each labeled image), our proposed method achieved competitive performance compared to the fully supervised pixel-wise segmentation models. Experiments demonstrate that the proposed solution has the potential to reduce the burden of labeling histopathological images.

## 1. Introduction

Deep learning has made rapid development and remarkable progress in pathological image analysis in recent years [1,2,3,4,5,6,7]. The application of deep learning in pathological diagnosis and prognosis cannot be imagined without high-quality annotations. However, acquiring precise annotations is difficult since it requires knowledge of pathology and is time-consuming and labor-intensive, particularly for segmentation tasks that involve manually outlining the specific structures.

Unfortunately, experts with a wealth of pathological knowledge, the source of high quality and clean clinical tagging of key data, are often scarce and have limited energy to spend on data labeling. Therefore, deep-learning methods based on sparsely annotated labels are critical to reducing their workload of labeling and pushing the application of deep learning in the field of pathology. Tumor segmentation has been one of the most fundamental tasks in digital pathology for accurate diagnosis.

Since a whole slide image (WSI) usually has an extremely high resolution, e.g., 50,000 × 50,000 pixels, common practice is to crop it into smaller images and assign each of them a label for model training. There are two typical models, including an image-wise segmentation model [8,9,10,11,12,13] and pixel-wise segmentation model [14,15,16,17,18]. An image-wise segmentation model predicts whether the given image contains tumorous regions.

A binary label (‘tumor’ or ‘normal’) is assigned to each image in the training set to train these models. However, the performance of an image-wise segmentation model is limited by the insufficiency of the labeling information. Since a mere binary label ‘tumor’ cannot reflect the location and proportion of the tumor, assigning the same label ‘tumor’ to different images as long as they contain the tumor may confuse the network training and lead to inaccurate segmentation results, which is unacceptable—in particular for small tumors.

In contrast, a pixel-wise segmentation model can produce more accurate segmentation results. However, pathologists must annotate the tumor regions as masks to train the model, which takes much more time and energy. More importantly, unlike other medical images, such as MRI and CT images, pathology images usually lack a clear distinction between the normal and tumor areas [19], which imposes additional difficulties for labeling.

**Figure 1:**
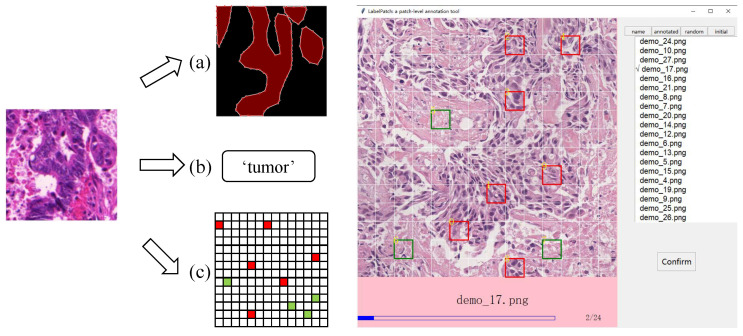
**Left:** The illustration of different types of labels. (**a**) Pixel-level label, where the red area denotes tumor region and the black area denotes non-tumor region. (**b**) Image-level label, suggesting that the image contains tumor region. (**c**) Patch-level label (proposed), where the red patches and green patches are manual annotations indicating tumor and non-tumor regions, respectively. **Right:** A software we developed for sparse patch annotation.

To compensate for the shortcomings of the above two methods, we propose the concept of the patch-level label. Note that, in our proposed method, a patch refers to a grid cell of an image, which is different from the definition in other articles [11,18]. Suppose we divide the image with the size of 224 × 224 pixels into a 14 × 14 grid, then the patch size is 16 × 16 pixels. For each image in the training set, pathologists only need to annotate several (usually 5–10) patches as the label, significantly saving the annotation cost. The left of Figure 1 shows different types of labels.

We designed a patch-wise segmentation model called Pseger to accommodate this new label. It has two branches for image classification and patch classification, respectively. The image classification is an auxiliary task that helps improve the performance of the patch classification branches. Due to the superior performance the Trasformer-based networks [20] have achieved in recent years, we select Swin Transformer [21], a representation of them as the backbone of the model. Moreover, this method can be easily extended to other backbones.

To take advantage of the unlabeled data, we trained our Pseger with an innovative semi-supervised algorithm. The algorithm is developed based on the characteristics of the patch-level label, integrating the ideas of consistent learning [22] and self-training [23]. The contributions of this paper are summarized as follows:

- We proposed the concept of sparse patch annotation for tumor segmentation, which can significantly reduce the annotation burden. To achieve this new way of labeling, we developed an annotation tool (Figure 1, right).
- In order to handle this new label, we created a patch-wise segmentation model called Pseger, which was equipped with an innovative semi-supervised algorithm to make full use of the unlabeled data.
- We comprehensively evaluated our proposed method on two datasets. The experimental results showed that when trained with only 25% labeled data (five patches for each labeled image), our approach can yield a competitive result compared to the pixel-wise segmentation models trained using 100% labeled data. The ablation study showed the effectiveness of the semi-supervised algorithm.

## 2. Related Works

### 2.1. Weakly-Supervised Learning

Pixel-level labels require a considerable amount of time and effort, and the frequently occurring manual errors may give the network the wrong guidance. Weakly-supervised learning (WSL) has recently emerged as a paradigm to relieve the burden of dense pixel-wise annotations [24]. Many WSL techniques have been proposed, including global image-level labels [25,26], scribbles [19,27], points [28,29], bounding boxes [30,31], and global image statistics, such as the target-region size [32,33].

Although these weakly supervised methods have achieved good performance in natural and medical image segmentation, most weak annotations may not necessarily be best or most suited for tumor segmentation. As mentioned above, the image-level label cannot reflect the location and proportion of the tumor, which may result in inaccurate segmentation results. Other label types are more suitable for segmentation tasks where the instances have clear boundaries, such as glands and nuclei. Nevertheless, the boundary between the normal and the tumor area in pathology images is usually fuzzy and ambiguous. Unlike existing weak annotations, we propose patch-level annotation for patch-wise tumor segmentation.

### 2.2. Multi-Task Learning

Multi-task learning is an emerging field in machine learning that seeks to improve the performance of multiple related tasks by leveraging useful information among them [34]. A deep-learning model for multi-task learning usually consists of a feature extractor shared by all the tasks and multiple branches for each task. In recent years, multi-task learning has been widely exploited in the field of pathological image analysis [18,35,36]. For example, Wang et al. [18] proposed a hybrid model for pixel-wise HCC segmentation of H&E-stained WSIs.

The model had three subnetworks sharing the same encoder, corresponding to three associated tasks. Guo et al. [37] employed a classification model to filter images containing tumorous regions and subsequently refined the segmentation results by a pixel-wise segmentation model. Inspired by these seminal works, we adopted a two-branch model, one branch for image classification and another for patch segmentation, to learn more general features and thus reduce the risk of overfitting.

### 2.3. Semi-Supervised Learning

Semi-supervised learning (SSL) is a combination of both supervised and unsupervised learning methods, in which the network is trained with a small amount of labeled data and a large amount of unlabeled data. SSL methods can make full use of the information provided by unlabeled data, thereby improving the model performance. In recent years, SSL methods have been widely used in the computer vision field [38,39,40,41,42,43].

There are two common SSL strategies, including consistent learning [22] and self-training [23]. The general idea of consistent learning is that model prediction should keep constant under different perturbations to the input. This method allows for various perturbations to be designed depending on the characteristics of the data and the network. For instance, Xu et al. [40] proposed two novel data augmentation mechanisms and incorporated them into the consistency learning framework for prostate ultrasound segmentation.

Another strategy, self-training, can be broadly divided into four steps. First, train a teacher model using labeled data. Second, use a trained teacher model to generate pseudo labels for unlabeled images. Third, learn an equal-or-larger student model on labeled and unlabeled images. Finally, use the student as a teacher and repeat the above procedures several times. Wang et al. [41] proposed a few-shot learning framework by combining ideas of semi-supervised learning and self-training. They first adopted a teacher-student model in the initial semi-supervised learning stage and obtained pseudo labels for unlabeled data. Then, they designed a self-training method to update pseudo labels and the segmentation model by alternating downsampling and cropping strategies.

## 3. Materials and Methods

**Figure 2:**
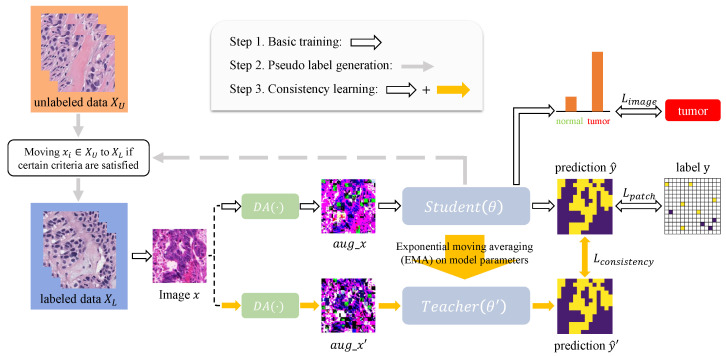
Overview of the framework for training PSeger. DA(·) indicates data augmentation module.

Here, we propose a novel patch-wise segmentation model called PSeger. Equipped with an innovative semi-supervised algorithm, it can learn from the patch-level label and take advantage of the unlabeled data. Figure 2 gives an overview of the training procedure. It involves three steps: (1) basic training; (2) pseudo label generation; and (3) consistency learning. They are described in detail in the following. The information about the two datasets we used is also described later.

### 3.1. Basic Training

**Figure 3:**
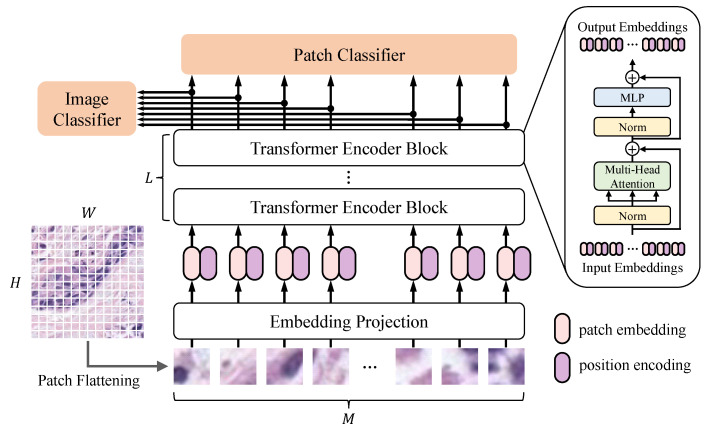
Illustration of PSeger (using Vistion Transformer as backbone).

Since the idea of patch-level label is inspired by Vision Transformer (ViT) [20], we take it as the backbone of PSeger to illustrate the process of basic training. An overview of the model is depicted in Figure 3, which consists of an embedding projection module, a sequence of transformer encoder blocks, and two classifiers for image classification and patch classification, respectively. In the process of forward propagation, an input image $x\in R^{H\times W\times N_{C}}$ (*H*, *W*, and $N_{C}$ represent the height, width, and number of channels of *x*, respectively) is first flattened into $M=HW/P^{2}$ non-overlapped patches with the size of P×P pixels. Then, a 2-D convolution operation is employed to obtain patch embeddings, supplemented with position encoding:

(1) $$z_{0}=\left[x^{1}P_{E};x^{2}P_{E};\ldots ;x^{M}P_{E}\right]+P_{E}^{pos},$$

where $z_{0}\in R^{M\times L}$ (*L* represents the embedding length) is the input of the first transformer encoder block, $x^{k}\in R^{P\times P\times C}$ is the *k*th patch, $P_{E}$ is the embedding projection, and $P_{E}^{pos}$ is the position encoding. Then, the embeddings are processed by the transformer encoder blocks. Each block includes a multi-head self-attention (MSA) [44] module and a multi-layer perceptron (MLP) module, both of which are operating as residual operators, and with a layer normalization (LN) [45]. The output of the *l*th transformer encoder block can be described as follows,

(2) $$z_{l}^{\prime }=MSA\left(LN\left(z_{l-1}\right)\right)+z_{l-1},\;\;l=1\ldots L,$$

(3) $$z_{l}=MLP\left(LN\left(z_{l}^{\prime }\right)\right)+z_{l}^{\prime },\;\;\;\;\;\;\;l=1\ldots L,$$

where $z_{L}$ is the final output of the transformer encoder. Each element of the output $z_{L}^{k}\in z_{L}$ contains contextual features due to the attention mechanism, which makes it possible to classify a patch based on the information of the related patches. We adopt an MLP head $H_{patch}$ for patch classification. By these means, $z_{l}$ processed by an LN is sent to $H_{patch}$ before applying a softmax function to obtain predictions of each patch:

(4) $$\hat{y}=Softmax\left(H_{patch}\left(LN\left(z_{L}\right)\right)\right),$$

where $\hat{y}\in R^{M\times C}$ are the patch predictions, and *C* is the number of categories.

In addition to the patch classifier, we introduce an auxiliary image classifier $H_{image}$ to the network, which determines whether an input image has a tumor or not. The main motivation for use of image classifier is to help the patch classifier achieve better performance, since in multi-task learning the network tends to find more representative features shared by different tasks [18]. Similar to the patch classifier, the image classifier receives the average of the *L*th transformer encoder output $z_{L}\in R^{M\times L}$ with an LN, and produces the classification result ${\hat{y}}_{img}\in R^{C}$ through a softmax function:

(5) $${\hat{y}}_{img}=Softmax\left(H_{image}\left(LN\left(\sum_{i=1}^{M}z_{L}^{k}/M\right)\right)\right).$$

The loss function for the basic training is defined as:

(6) $$L_{sup}=L_{patch}+\alpha L_{img},$$

where $L_{img}$ and $L_{patch}$ are the losses for image classification task and patch classification task, respectively. α is a weighting factor for the two losses. Both $L_{img}$ and $L_{patch}$ are cross-entropy loss functions; however, $L_{patch}$ only considers the annotated patches. Specifically, $L_{patch}$ is defined as:

(7) $$L_{patch}=-\frac{1}{K}\sum_{k}^{K}\sum_{c}^{C}y^{k}\log{\hat{y}}^{\left(k,c\right)},$$

where *K* is the number of the labeled patches in the sample *x*, *C* is the number of classes, $y^{k}$ is the binary indicator (0 or 1) if class label *c* is the correct classification for the *k*th patch. ${\hat{y}}^{\left(k,c\right)}$ is the prediction of the *k*th patch at the *c*th class.

### 3.2. Pseudo Label Generation

**Figure 4:**
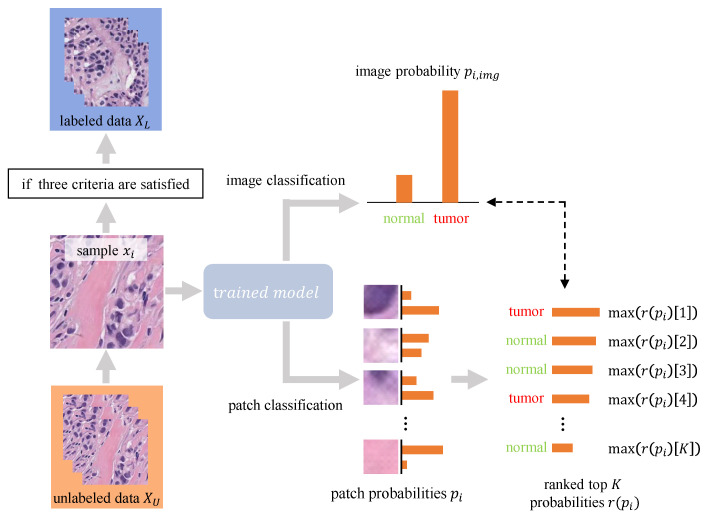
Illustration of the pseudo label generation process. Note that the ranked top *K* probabilities $r(p_{i})$ only displays the dominant values for each $r\left(p_{i}\right)\left[k\right],k\in \left[1,K\right]$. For example, if $r\left(p_{i}\right)\left[k\right]$ is ’tumor’: 0.6, ’normal’: 0.4, then the dominant value of $r\left(p_{i}\right)\left[k\right]$ is ’tumor’: 0.6. Thus, $max(r\left(p_{i}\right)\left[k\right])=0.6$ and $argmax(r\left(p_{i}\right)\left[k\right])=$ ’tumor’.

After the basic training process, the model with the best patch classification accuracy on the validation set is used to generate the pseudo labels for samples in the unlabeled data $X_{U}$, as is depicted in Figure 4. The trained model receives as input an image $x_{i}\in X_{U}$ and infers the image prediction ${\hat{y}}_{i,img}$ and patch predictions ${\hat{y}}_{i}$, which are subsequently transformed into the image probability $p_{i,img}$ and patch probabilities $p_{i}$ by the softmax function. The latter are then ranked by their dominant values. We move $x_{i}$ from $X_{U}$ to $X_{L}$ along with its pseudo label if $p_{i,img}$ and ranked $p_{i}$ (denoted as $r(p_{i})$) meet the following criteria:

1. $\max\left(p_{i,img}\right)>\tau_{1}$, where $\tau_{1}$ is the confidence threshold for the image prediction.
2. $\max\left(r\left(p_{i}\left[K\right]\right)\right)>\tau_{2}$, where $\tau_{2}$ is the confidence threshold for the patch prediction.
3. ∀k∈[1,K],$\mathrm{argmax}\left(r\left(p_{i}\right)\left[k\right]\right)=\mathrm{argmax}\left(p_{i,img}\right)$, which means the patch predictions should remain consistent with the image prediction.

We made some attempts with small-scale data in the early stage and found that the image prediction confidence scores were high (usually above 0.9); however, the patch prediction confidence scores were relatively low (usually below 0.7). Therefore, we empirically set $\tau_{1}$ to 0.8 and $\tau_{2}$ to 0.6.

### 3.3. Consistency Learning

When the step of pseudo label generation is finished, the model begins to retrain on the updated training set $X_{L}$. The details are as follows. First, for an input image $x\in X_{L}$, it is transformed into aug_x and $aug\_x^{\prime }$ by twice independent data augmentation operation. Then, the student model and the teacher model take them as input and output two sets of patch predictions $\hat{y}$ and ${\hat{y}}^{\prime }$, respectively. These two sets should remain consistent based on the smoothness assumption in semi-supervised learning [46]. Therefore, we apply the KL divergence consistency loss between $\hat{y}$ and ${\hat{y}}^{\prime }$:

(8) $$L_{cons}=-\frac{1}{M}\sum_{m}^{M}\sum_{c}^{C}{\hat{y}}^{\left(m,c\right)}\;\log\frac{{\hat{y}}^{\left(m,c\right)}}{{{\hat{y}}^{\prime }}^{\left(m,c\right)}}.$$

where *M* is the number of patches in the sample *x*; *C* is the number of categories; ${\hat{y}}^{\left(m,c\right)}$ and ${{\hat{y}}^{\prime }}^{\left(m,c\right)}$ are the predictions of the *m*th patches at the *c*th category. Thus, the total loss function can be written as,

(9) $$L_{total}=L_{sup}+\lambda \left(E\right)L_{cons},$$

where $L_{sup}$ is previously defined in Equation (6). $\lambda \left(E\right)$ is a function of training epoch index *E*, which helps control the balance between the supervised loss and the consistency loss. As is the case with other consistency learning methods [40,47], we use a Gaussian ramp-up function as $\lambda \left(E\right)$:

(10) $$\lambda \left(E\right)=\left\{\begin{array}{cc} \lambda_{max}\;\cdot \;exp\left[-5{\left(1-\frac{E}{E_{max}}\right)}^{2}\right], & E<E_{max}\\ \lambda_{max}, & \mathrm{otherwise} \end{array}\right.,$$

where *E* is the epoch index. When $E=E_{max},\;\lambda$ reaches the maximum weight $\lambda_{max}$ for the consistency loss. We empirically set $\lambda_{max}$ to 1 and $E_{max}$ to 20 epochs. For the student model, the parameters θ are updated through back-propagation algorithm by minimizing $L_{total}$. For the teacher model, the parameter $\theta^{\prime }$ are initially set to $\theta_{0}$ and updated by computing the exponential moving average of θ:

(11) $$\theta_{t}^{\prime }=\alpha \theta_{t-1}^{\prime }+\left(1-\alpha \right)\theta_{t}.$$

where *t* represents the index of the global training steps. α helps control the speed at which the teacher model parameters $\theta^{\prime }$ are updated, and we empirically set it to 0.99.

### 3.4. Datasets

We evaluated our proposed method on a public dataset BCSS [48] and an in-house dataset. BCSS dataset includes 151 hematoxylin and eosin-stained images corresponding to 151 histologically-confirmed breast cancer cases. The mean image size is 1.18 mm${}^{2}$ (SD = 0.80 mm${}^{2}$). We followed the train-test splitting rule (https://bcsegmentation.grand-challenge.org/Baseline/ (accessed on 1 June 2022) ) that the images from these institutes were used as an unseen testing set to report accuracy: OL, LL, E2, EW, GM, and S3. (The abbreviations stand for tissue source sites (For more details, see https://docs.gdc.cancer.gov/Encyclopedia/pages/TCGA_Barcode/) (accessed on 1 June 2022)). Then, the remained 108 images were cropped into 27,207 smaller images (with the size of 224 × 224). We used 1018 of these smaller images for validation and the remained were for training.

The in-house dataset came from Department of Pathology, the First Affiliated Hospital of Sun Yat-sen University, China. This study was approved by the Ethics Committee of First Affiliated Hospital of Sun Yat-sen University, and data collection were performed in accordance with relevant guidelines and regulations. The dataset contains 28,187 images from 111 cases (WSIs). We used the images of 84 cases for training and validation, and the images from the remaining cases for test. For the training set, 292 images were from the non-tumor regions, labeled as ‘normal’.

A total of 24,971 images were from tumor regions but many of them did not contain any tumor cells. We selected 407 out of these images and labeled 10 patches for each images using our self-developed annotation tool. Among these labeled images, if one contains any tumor cells, then at least one patch will be labeled as ‘tumor’, and the image label will be ‘tumor’, as well. Details about the BCSS dataset and the in-house dataset are shown in Table 1 and Table 2, respectively.

## 4. Results

### 4.1. Experimental Setup

#### 4.1.1. Training Settings

In the training step, we employed the AdamW optimizer [49] with a base learning rate of $5\times 10^{-4}$. For the learning rate schedule, we adopted a linear warmup for five epochs (the warmup learning rate was $5\times 10^{-7}$), followed by cosine annealing for 20 epochs. The batch size was 16, and the backbones used for Pseger were pre-trained on ImageNet. All experiments were done with a RTX 3090. There are five training strategies for PSeger:

- *Baseline:* train the model only on the labeled data.
- *Baseline+CL:* train the model only on the labeled data with consistency learning.
- *Baseline+CL with $X_{u}$:* train the model on both the labeled data and unlabeled data with consistency learning.
- *Baseline+ST with $X_{u}$:* first train the model on the labeled data, then use the trained model to infer the pseudo labels of the unlabeled data, and finally retrain the model on both the labeled data and pseudo-labeled data.
- *Baseline+ST+CL with $X_{u}$:* first train the model on the labeled data, then use the trained model to infer the pseudo labels of the unlabeled data, and finally retrain the model on both the labeled data and pseudo-labeled data with consistency learning.

#### 4.1.2. Evaluation Metrics

In the experiment of comparison with segmentation models, we choose Intersection over Union (IoU) as the evaluation indicator, which is calculated as follows,

(12) $$\mathrm{IoU}=\frac{A\cap B}{A\cup B},$$

where A and B are the predicted tumor area and ground truth, respectively. The final IoU score is obtained by averaging the IoU for each RoI in the BCSS test set.

In the ablation study, since our in-house dataset has no pixel-wise annotations, we select patch-level and image-level Acc, AUC, and F1 as evaluation indicators. AUC (Area Under the Curve) score is simply the area under the Receiver Operating Characteristic (ROC) curve. Acc and F1 are calculated as follows,

(13) $$\mathrm{Acc}=\frac{\mathrm{TP}+\mathrm{TN}}{\mathrm{TP}+\mathrm{TN}+\mathrm{FP}+\mathrm{FN}},$$

(14) $$F1=\frac{2\mathrm{TP}}{2\mathrm{TP}+\mathrm{FP}+\mathrm{FN}},$$

where TP, TN, FP, and FN are true positive, true negative, false positive, and false negative, respectively. The final scores of each evaluation indicator are calculated by averaging the score for each image in BCSS or the in-house test set.

### 4.2. Comparison with Segmentation Models

**Figure 5:**
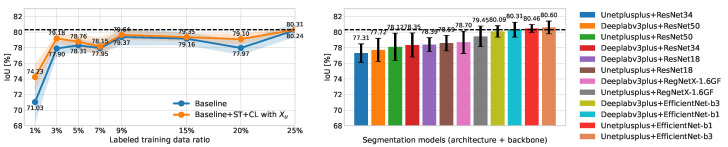
Comparison between our proposed method and pixel-wise segmentation models on the BCSS dataset. **Left:** IoU values of PSeger trained on different ratios of labeled training data by two training strategies (*Baseline*, *Baseline+ST+CL with $X_{u}$*). **Right:** IoU values of different segmentation models trained on the full training set. The values of the black dotted lines in the left and right are both 80.31, representing the IoU that PSeger (trained by *Baseline+ST+CL with $X_{u}$* on the training set with 25% labeled data) and the third-best segmentation model (DeepLabv3plus+EfficientNet-b1) have achieved.

We compared our proposed method to a variety of segmentation models on the BCSS dataset (Figure 5). We trained PSeger with two strategies: *Baseline* and *Baseline+ST+CL with $X_{u}$*. Five patches were labeled for each images in the labeled training set, and ratios of labeled training data were from 1% to 25%. In comparison, we chose two architectures of segmentation models, DeepLabv3+ [50] and Unet++ [51], and equipped them with six backbones: ResNet18, ResNet34, ResNet50 [52], EfficientNet-B1, EfficientNet-B3 [53], and RegNetX-1.6GF [54], respectively.

Therefore, 12 segmentation models were trained and tested on the BCSS dataset. These segmentation models and the training and test steps were implemented base on SegmentationModels [55]. By comparing the two graphs in Figure 5, we can see that when the proportion of labeled training data reaches 25%, our proposed method can achieve 80.31 ± 0.23% IoU on the test set, comparable with the third-best model (DeepLabv3plus+EfficientNet-b1: IoU = 80.31 ± 0.95%) out of 12 segmentation models.

### 4.3. Visualization of Segmentation Results

**Figure 6:**
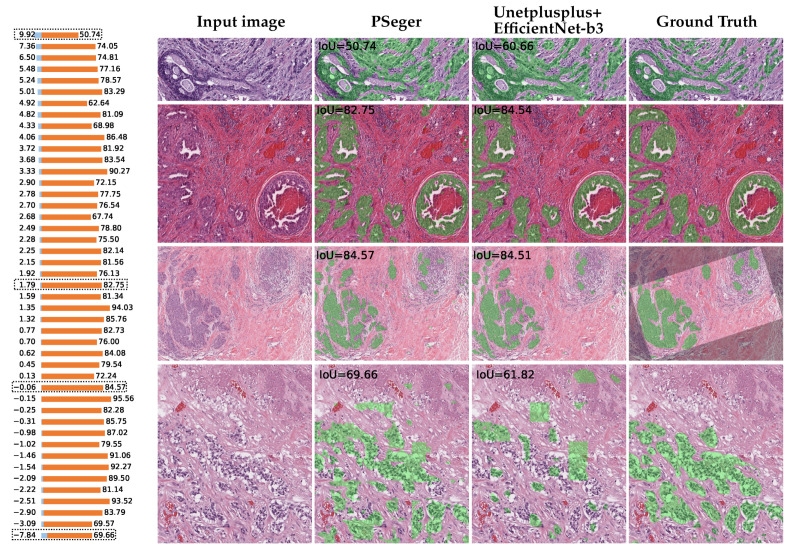
Comparison between PSeger and Unetplusplus+EfficientNet-b3. **Left:** IoU values (orange bars) of PSeger on 45 tested ROIs and their differences (sky-blue bars) with those of Unetplusplus+EfficientNet-b3. The bar pairs are sorted in descending order of the values of the blue bars. **Right:** Images of four representative cases. From top to bottom, rows are case 1–4, also framed by black dotted rectangles in the bar graph on the left. From left to right, columns are input images, segmentation results by PSeger, segmentation results by Unetplusplus+EfficientNet-b3, and ground truths. Green overlays are annotated or predicted tumor regions, black overlays are ignored regions, and others are non-tumor regions.

To further compare our proposed method with the pixel-wise segmentation method, we selected one of the best performing PSegers (trained by *Baseline+ST+CL with $X_{u}$* with 25% images in the training set labeled, IoU = 80.65%) and compared it with the best performing model in segmentation models (Unetplusplus+EfficientNet-b3, IoU = 81.74%), as is shown in Figure 6.

In general, the performance of PSeger is comparable to that of Unetplusplus+EfficientNet-b3. The largest prediction differences aroused in case 1 and case 4. In case 1, PSeger performed worse because of more false detection on non-tumorous area; in case 4, Unetplusplus+EfficientNet-b3 performed poorly because of more false positive regions and much more missed detection on tumorous area.

**Figure 7:**
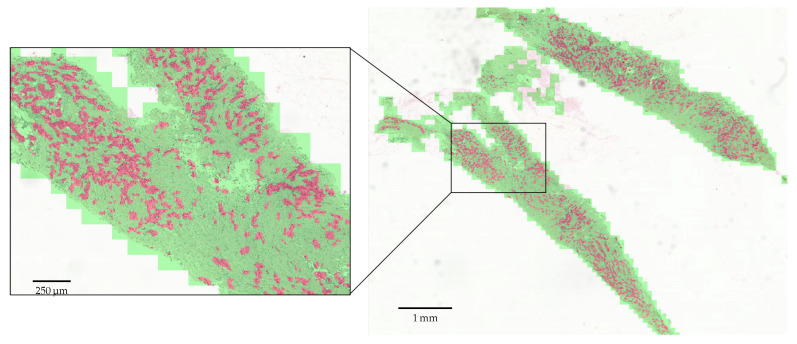
Segmentation results on a whole slide image.

**Figure 8:**
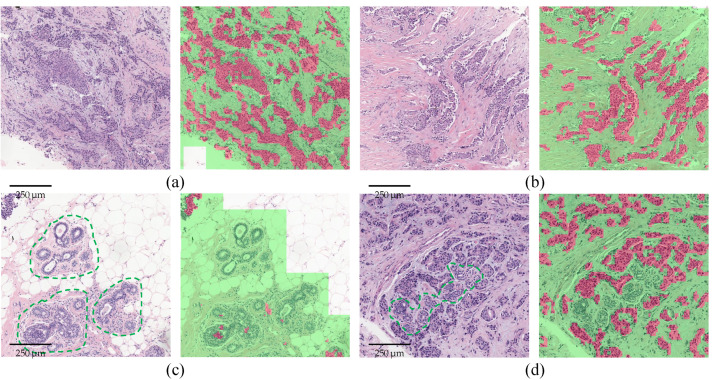
Segmentation results of some ROIs. (**a**,**b**) Examples of invasive tumor. (**c**) An example of lobules (a normal structure in breast tissue). (**d**) An example of lobules surrounded by the invasive tumor. Lobules in (**c**,**d**) are outlined by green dashed polygons.

In addition, Figure 7 and Figure 8 display some segmentation results on our in-house dataset. Red and green overlays are tumor regions and non-tumor regions judged by PSeger, respectively, while regions not covered by any overlay are background areas. It can be seen from Figure 8 that our method can accurately segment the invasive tumor and distinguish some non-tumor structures easily confused with tumors.

### 4.4. Ablation Study

#### 4.4.1. The Effect of the Amount of Labeling

**Figure 9:**
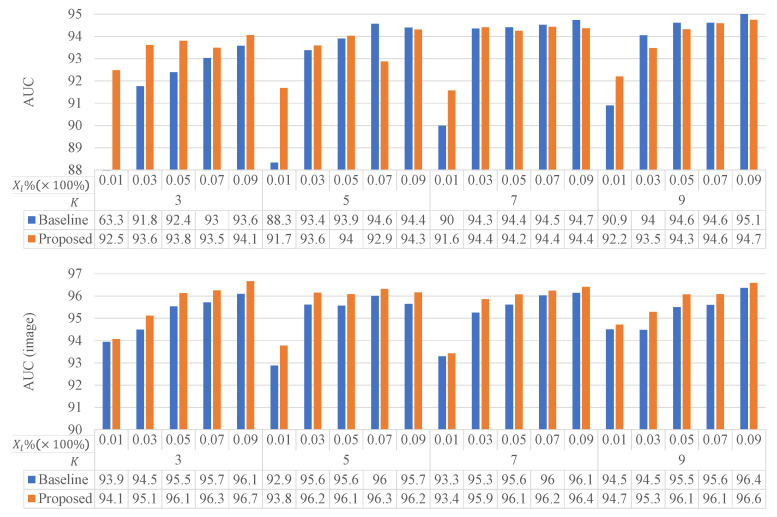
The effect of the amount of labeling.

As an important factor affecting model performance, the amount of labeling is reflected in two aspects: the ratio of annotated training samples to all training samples (denoted as $X_{l}\%$), and the number of the labeled patches in each sample (denoted as *K*). We conducted experiments on the BCSS dataset to examine the effect of $X_{l}\%$ and K on the model performance. Figure 9 shows the patch-level AUC values and the image-level AUC values of Baseline and Baseline+ST+CL with $X_{u}$ under different $X_{l}\%$ and *K*, respectively, and the results are given as the mean of three experiments performed in duplicate.

Overall, the two AUC values have increased with increased $X_{l}\%$ and *K*. However, the increase has slowed down with higher $X_{l}\%$ and K. More importantly, Baseline+ST+CL with $X_{u}$ always outperforms Baseline on image-level AUC, while the former has better patch-level AUC than the latter only when $X_{l}=1\%$ or K=3.

#### 4.4.2. Training with Different Strategies

To assess the contributions of self-training and consistency learning separately, we performed experiments on the BCSS dataset and the in-house dataset with five different training strategies mentioned before. Each experiment was repeated five times independently and the results are summarized in Table 3 and Table 4, where bold and underlined values represent the best and second-best results on a metric, respectively.

From Table 3, the strategy of Baseline+ST+CL with $X_{u}$ helps PSeger achieve the best performance on four of the six indicators (AUC = 92.04%, Acc = 85.72%, F1 = 80.4%, AUC${}_{img}$ = 94.31%), significantly higher than the value that the strategy of Baseline has achieved (AUC = 88.62%, Acc = 84.28%, F1 = 78.63%, AUC${}_{img}$ = 93.25%). The strategy of Baseline+ST with $X_{u}$ achieves the second-best performance (AUC = 91.98%, Acc = 85.58%, F1 = 80.05%, AUC${}_{img}$ = 94.05%), which is roughly similar to that of Baseline+ST+CL with $X_{u}$. Additionally, the performance of Baseline+CL is inferior to that of Baseline. Furthermore, when $X_{u}$ is involved in the training procedure, the model (Baseline+CL with $X_{u}$) performs better than Baseline and has reached the highest in the two indicators of Acc${}_{img}$ (86.17%) and F1${}_{img}$ (87.55%).

From Table 4, while the performance of PSeger trained by Baseline+ST+CL with $X_{u}$ on the in-house dataset is still better than that trained by Baseline, combining the two semi-supervised strategies (consistency learning and self-training) does not achieve better performance than either.

#### 4.4.3. Backbone Selections

In this experiment, we used all labeled data in the BCSS training set to train the models with different backbones, including DenseNet121 [56], EfficientNet-B0, EfficientNet-B1 [53], HRNet-w18 [57], ResNet18, ResNet34, ResNet50 [52], ResNeXt-101 (32 × 8d) [58], ViT-base [20], and Swin-base [21] and tested their performance on the BCSS test set (Table 5). The experiment was repeated five times. From the results, the model using Swin-base as backbone achieves the best performance, significantly better than other models.

Nevertheless, the CNN-based models still achieve decent outcomes. It is somewhat surprising that the model using ViT-base as the backbone is not as good as the models using the CNN architecture in the patch-level evaluation indexes; however, it can surpass most CNN architecture models in the image-level evaluation indexes (second only to ResNeXt-101 (32 × 8d)).

## 5. Discussion

In the ablation study, we first investigated the effect of the amount of labeling on model performance (Figure 9). On the image-level AUC, the model trained by *Baseline+ST+CL with $X_{u}$* was always better than that trained by *Baseline* under otherwise equal conditions. However, on the patch-level AUC, that was not always true, particularly when K>3 and $X_{l}\%>1\%$. This meant that the proposed semi-supervised method can effectively improve the image classification performance; however, it enhanced the patch classification performance only when the amount of annotation was small. When the annotation amount increased, the semi-supervised learning method was not as good as the fully-supervised learning method. Further study is therefore needed to optimize semi-supervised training.

Next, we performed experiments on different training strategies (Table 3 and Table 4). Both consistency learning and self-training benefited the model, and self-training improved the model performance more significantly. Additionally, combining the consistency learning strategy with the self-training strategy has the potential to fully utilize the pseudo-annotated data and further improve model performance. However, it depends on the dataset and requires appropriate parameter settings to achieve the expected result.

Finally, the experiment of training with different backbones (Table 5) proves that our proposed method is suitable for transformer-based models and models with CNN architecture. By comparing the performance of different models, we found that Swin Transformer was better than CNN models on both image-level metrics and patch-level metrics.

In comparison, Vision Transformer was only better than most CNNs on image-level metrics and inferior to many CNNs on patch-level metrics. This may because the patch classification accuracy depends on the ability to capture localized features and the sensitivity to context-driven features. Although Vision Transformer is more sensitive to contextual features than CNN models, its local feature extraction ability is poorer, which affects the final patch classification accuracy.

Our proposed method can be improved in several ways:

- **Hierarchical patch-level label.** Here, we only considered the annotation form at a single scale, which did not take advantage of the information at different magnifications of the pathological images. Therefore, the annotation can be extended to multiple scales, allowing the model to learn from hierarchical information.
- **Automatic patch selection for labeling.** Choosing which patches to label is subjective and will affect the learning effect of the model. Hence, an active learning mechanism [59] can be introduced to automatically find the most informative patches to label, improving learning efficiency.
- **Hybrid CNN-transformer architecture.** In terms of local feature extraction and global feature capture, CNN and transformer have respective advantages, as analyzed before. Therefore, a hybrid CNN-transformer architecture, like in [60,61], might combine the benefits of the two better to achieve greater performance.
- **More advanced semi-supervised algorithm.** Our semi-supervised algorithm still has problems, such as being sensitive to hyperparameters. In the future, ideas from some advanced semi-supervised algorithms in recent years, such as Mixmatch [62], can be introduced into the training algorithm. At the same time, some constraints can be added to prevent the model from overfitting, such as the consistency of prediction results between the patch classification branch and the image classification branch.

## 6. Conclusions

In this work, we proposed a novel form of annotation, sparse patch annotation, and developed an annotation tool to achieve this new way of labeling. We created a patch-wise segmentation model called Pseger to handle this new label, which was equipped with an innovative semi-supervised algorithm to fully utilize the unlabeled data. We compared the proposed method to various pixel-wise segmentation models (Figure 5). It was shown that, when trained with only 25% labeled data (five patches for each labeled image), our model achieved comparable segmentation results with the semantic segmentation models trained on fully pixel-level labeled data.

Our proposed method enables pathologists to focus their time and energy on labeling the representative parts of the image rather than carefully delineating complex boundaries, significantly reducing the annotation burden.

## Figures and Tables

**Table 1:** Summary of the BCSS dataset.

Cases/WSIs/ROIs	151
ROIs for training and validation	106
Images (224 × 224) for training	26,189
Images (224 × 224) for validation	1018
ROIs for test	45
Images (224 × 224) for test	9444

**Table 2:** Summary of the in-house dataset.

Cases/WSIs	111
Cases for training and validation	84
Patch-level-labeled Images (224 × 224) for training	407
Patch-level-labeled Images (224 × 224) for validation	292
Images (224 × 224) from non-tumor regions for training	222
Unlabeled images (224 × 224) for training	24,564
Cases for test	27
Patch-level-labeled Images (224 × 224) for test	2702

**Table 3:** Model performance on the BCSS dataset with different training strategies.

Training Strategy	AUC	Acc	F1	AUC${}_{\mathrm{img}}$	Acc${}_{\mathrm{img}}$	F1${}_{\mathrm{img}}$
*Baseline*	88.62±0.99	84.28±0.68	78.63±1.44	93.25±0.71	85.91±0.62	87.21±0.67
*Baseline+CL*	88.41±1.20	83.71±1.43	77.62±2.45	93.23±0.84	86.06±0.63	87.41±0.70
*Baseline+CL with $X_{u}$*	88.67±0.82	84.02±0.74	78.29±1.68	93.09±0.81	86.17±0.59	87.55±0.61
*Baseline+ST with $X_{u}$*	91.98±0.49	85.58±0.57	80.05±1.28	94.05±0.74	85.48±1.57	86.39±1.83
*Baseline+ST+CL with $X_{u}$*	92.04±0.36	85.72±0.65	80.40±1.53	94.31±0.32	85.89±1.49	86.85±1.75

**Table 4:** Model performance on the in-house dataset with different training strategies.

Training Strategy	AUC	Acc	F1	AUC${}_{\mathrm{img}}$	Acc${}_{\mathrm{img}}$	F1${}_{\mathrm{img}}$
*Baseline*	89.73±0.60	81.79±0.61	82.98±0.47	97.07±0.72	92.28±0.76	92.36±0.74
*Baseline+CL*	89.92±0.52	81.96±0.41	83.17±0.27	96.57±0.67	92.46±0.62	92.53±0.61
*Baseline+CL with $X_{u}$*	89.64±0.24	82.12±0.58	83.28±0.4	96.70±1.07	92.84±0.27	92.90±0.26
*Baseline+ST with $X_{u}$*	90.26±0.45	82.97±0.52	83.9±0.37	97.11±1.03	92.65±0.42	92.72±0.41
*Baseline+ST+CL with $X_{u}$*	89.26±0.74	82.14±0.4	83.22±0.4	96.78±0.42	92.86±0.26	92.92±0.25

**Table 5:** Model performance on the BCSS dataset using different backbones.

Backbone	AUC	ACC	F1	AUC (Image)	ACC (Image)	F1 (Image)
DenseNet121	94.33±0.06	87.47±0.09	83.04±0.13	95.68±0.12	89.67±0.22	91.22±0.20
EfficientNet-B0	94.76±0.12	87.57±0.19	83.00±0.37	95.66±0.08	89.57±0.24	91.14±0.18
EfficientNet-B1	94.57±0.04	87.30±0.04	82.71±0.17	95.80±0.10	89.60±0.07	91.15±0.02
HRNet-w18	94.31±0.09	87.21±0.14	82.47±0.27	95.99±0.10	89.99±0.18	91.39±0.14
ResNet18	94.03±0.09	87.04±0.12	82.26±0.21	95.35±0.19	88.96±0.26	90.56±0.22
ResNet34	94.35±0.05	87.37±0.09	82.85±0.16	95.72±0.16	89.62±0.28	91.17±0.21
ResNet50	94.11±0.12	87.33±0.13	82.76±0.29	95.94±0.18	90.01±0.38	91.48±0.30
ResNeXt-101 (32 × 8d)	94.64±0.09	87.58±0.09	83.11±0.13	96.25±0.07	90.34±0.14	91.64±0.09
ViT-base	94.47±0.07	87.39±0.06	82.94±0.08	96.16±0.09	90.20±0.21	91.66±0.17
Swin-base	95.41±0.05	88.40±0.08	84.29±0.12	96.64±0.10	91.47±0.04	92.70±0.05

## Data Availability

Our annotation tool is available at: https://github.com/FHDD/PSeger-LabelMe (accessed on 1 June 2022). The public dataset used in this study can be accessed at the following link: https://bcsegmentation.grand-challenge.org/ (accessed on 1 June 2022). The private dataset is available upon reasonable request to the corresponding authors.

## Abbreviations

The following abbreviations are used in this manuscript:

AUC	Area Under the Curve
BCSS	Breast Cancer Semantic Segmentation
CT	Computed Tomography
CL	Consistency Loss
HCC	Hepatocellular Carcinoma
H&E	Hematoxylin and Eosin
IoU	Intersection over Union
LN	Layer Normalization
MLP	Multi-layer Perceptron
MRI	Magnetic Resonance Imaging
MSA	Multi-head Self-attention
ROC	Receiver Operating Characetristic
ROI	Region of Interests
SD	Standard Deviation
SSL	Semi-supervised Learning
ST	Self-training
ViT	Vision Transformer
WSI	Whole Slide Image
WSL	Weakly-supervised Learning
